# Oral Hygiene Behaviors and Their Association with Angle Malocclusion Classes in Children Aged 6–9 Years: A WHO Questionnaire-Based Study

**DOI:** 10.3390/healthcare14131837

**Published:** 2026-06-24

**Authors:** Kaltrina Veseli, Fehim Haliti, Enis Veseli

**Affiliations:** 1 Department of Orthodontics, Alma Mater Europaea, Campus College Rezonanca, 10000 Prishtina, Kosovo; 2 Department of Pediatric and Preventive Dentistry, Dental School, Faculty of Medicine, University of Prishtina, 10000 Prishtina, Kosovo; 3 Department of Prosthodontics, Dental School, Faculty of Medicine, University of Prishtina, 10000 Prishtina, Kosovo

**Keywords:** oral hygiene, malocclusion, Angle classification, WHO Oral Health Questionnaire, children, oral-health-related quality of life, preventive dentistry, pediatric oral health

## Abstract

Background: Childhood oral hygiene behaviors are crucial to preventing oral diseases and can influence the development and progression of malocclusions. The World Health Organization (WHO) Oral Health Questionnaire is a standardized tool for assessing oral hygiene behaviors, oral health-related behaviors, and preventive dental awareness in children. Aim: This study aimed to assess oral hygiene behaviours and examine associations between WHO Oral Health Questionnaire variables and Angle malocclusion classes among children aged 6–9 years. Materials and Methods: This cross-sectional study included 90 children aged 6–9 years from the Pristina region, Kosovo. Data were collected using the WHO Oral Health Questionnaire for Children, which assessed oral hygiene habits, toothbrushing frequency, fluoride awareness, dental attendance, dietary behaviors, oral symptoms, and oral-health-related quality of life. Malocclusion was classified according to Angle classification into Class I, II, and III malocclusions with 3D intraoral scanners, Aerolscan 3. Descriptive statistical analysis, Chi-square (χ^2^) test, Spearman correlation analysis, and reliability analysis using Cronbach’s Alpha were performed using SPSS Statistics 23.0 (IBM Corp., Armonk, NY, USA) and Statistica 7.1 (StatSoft Inc., Tusla, OK, USA). Results: Most participants reported regular oral hygiene practices, with 46.7% brushing their teeth two or more times daily. However, limited awareness regarding fluoride-containing toothpaste was observed, as most children answered “don’t know” regarding fluoride use. Occasional toothache or oral discomfort was reported by 33.3% of participants, while 23.3% reported dissatisfaction with dental appearance. Difficulty biting hard foods was present in 34.4% of children. Reliability analysis of the Q10 section demonstrated moderate internal consistency (Cronbach’s Alpha = 0.500). Chi-square analysis demonstrated no statistically significant association between Angle malocclusion classes and WHO questionnaire variables (*p* > 0.05). The highest χ^2^ value was observed for tooth-cleaning frequency (Q7) (χ^2^ = 11.97; *p* = 0.152), although the association remained statistically non-significant. Psychosocial impact questions and oral health-related quality of life questions also demonstrated no statistically significant association with malocclusion classes. Conclusions: oral hygiene practices, preventative oral health practices, and oral health-related experiences were comparatively similar among children in different Angle malocclusion classes. Although there were no statistically significant correlations found between malocclusion classes and WHO questionnaire variables, the results show that some children have psychosocial concerns about their dental appearance and insufficient awareness of preventive oral health. The WHO Oral Health Questionnaire is a useful epidemiological tool for evaluating pediatric oral health behaviors and may help build youth orthodontic and preventive oral health policies.

## 1. Introduction

Malocclusion is a common developmental condition characterized by an abnormal relationship between the maxillary and mandibular dental arches, resulting in deviations from normal occlusion. It represents one of the most prevalent oral health problems worldwide, after caries and periodontal disease. with reported prevalence rates ranging between 39% and 93% in children and adolescents [1,2]. Malocclusion, defined by the World Health Organization as a handicapping dentofacial anomaly, refers to abnormal occlusion and/or disturbed craniofacial relationships. These abnormalities can impact aesthetic appearance, function, facial harmony, and psychosocial well-being [3,4]. Beyond its effects on occlusion and dental alignment, malocclusion may influence oral-health-related quality of life. Children with malocclusion may experience functional limitations, difficulties in mastication, concerns regarding dental appearance, and psychosocial challenges that can affect daily activities and well-being [5]. Although malocclusion is primarily determined by genetic and developmental factors, its clinical manifestations may influence oral health experiences and oral hygiene behaviors. Difficulties in chewing, concerns about dental appearance, and oral discomfort may affect children’s daily oral health practices and their perception of oral health [6]. The World Health Organization (WHO) Oral Health Questionnaire for Children has been widely used to assess oral health behaviors, oral symptoms, and self-perceived oral health in pediatric populations. However, limited evidence is available regarding the distribution of these WHO questionnaire variables across different Angle malocclusion classes. Therefore, assessing schoolchildren’s dental health and oral hygiene is crucial since this developmental stage is crucial for forming lifetime oral health habits [7]. A population’s oral health status is influenced by many variables, including the frequency of routine dental checkups, toothbrushing, proximal cleaning device use, sugar intake, fluoride product consumption, and fluoridated water consumption [8,9,10]. Another aspect is the impact of a soft diet, which exacerbates oral hygiene maintenance by causing anterior teeth crowding and mouth breathing due to a narrower airway [11,12]. The condition of the teeth, periodontium, and oral-facial system is commonly referred to as oral health [13]. Tooth decay, discomfort, poor academic performance, mood swings, low self-esteem, and a lower quality of life are all consequences of poor oral health. Eating, speaking, smiling, and expressing emotions are all made possible by having healthy teeth [14]. Increasing awareness of the value of oral hygiene and continued care is essential to preventing dental caries in youngsters. Teachers and parents are crucial to this process. Early oral health education and understanding are the first steps in the formation of these habits [15]. According to studies, dental issues like periodontitis may worsen mental health issues like anxiety and depression by causing inflammation in the body. Untreated mental health issues can also result in a lack of dental care and oral hygiene, which can lead to a vicious cycle of declining health [16]. Enhancing dental health boosts self-esteem and lessens social isolation, both of which are frequently associated with mental health issues [17]. By combining professional care with self-care, oral health conditions resulting from gum disease and teeth can be avoided. To evaluate oral hygiene practices, oral health awareness, and access to dental treatment among children and adolescents worldwide, the World Health Organization (WHO) created standardized oral health questionnaires. These surveys enable comparisons between people from various nations and areas and offer useful epidemiological data. Additionally, WHO-based evaluations aid in the formulation of preventive public health initiatives and help identify behavioral risk factors linked to poor oral health outcomes [18]. Maintaining good dental cleanliness, utilizing fluoride products, consuming fewer refined carbohydrates, and routinely visiting the dentist for expert preventative treatment, routine examinations, and oral health education are all components of proper oral hygiene [19]. Toothbrushing is considered the most common and effective method for maintaining oral hygiene. Personal oral care practices, such as regular toothbrushing and interdental cleaning, play a significant role in maintaining oral health and preventing oral diseases by removing dental plaque and reducing its accumulation on the teeth and gingival tissues [20,21,22]. Furthermore, data have shown the impact of malocclusion and orthodontic appliances on periodontal health; consequently, some patients are choosing aesthetic methods, such as aligners, to treat malocclusion, although their use has recently been controversial [23,24,25]. However, tooth brushing techniques are also of great importance, as inadequate techniques may not effectively remove dental plaque and can additionally contribute to enamel wear and gingival recession. For this reason, adequate knowledge regarding oral hygiene practices, products, and behaviors is essential for the prevention of oral diseases and the maintenance of optimal oral health [20,26,27]. Children aged 6–9 years represent an important developmental period characterized by mixed dentition, craniofacial growth, and the establishment of long-term oral hygiene habits [28]. At this stage, inadequate oral hygiene practices may contribute not only to dental diseases but also to the development or aggravation of malocclusions and other orthodontic problems. Previous studies have shown that children’s oral hygiene behaviors are influenced by multiple factors, including parental education [6,29], demographic and socioeconomic factors, which then significantly influence the prevalence and development of dental caries. Research shows that children and adolescents from disadvantaged socioeconomic backgrounds are more likely to experience dental caries due to reduced access to dental services, poorer oral hygiene habits that negatively affect dietary choices [30]. Although WHO oral health questionnaires are widely used for evaluating oral hygiene behaviors and oral-health-related quality of life in pediatric populations, limited evidence exists regarding their association with Angle malocclusion classes in mixed dentition children assessed using contemporary digital intraoral scanning methods. Furthermore, data from Southeast European pediatric populations, particularly Kosovo, remain scarce. Understanding the relationship between oral hygiene behaviors, psychosocial oral-health experiences, and malocclusion patterns during early mixed dentition may contribute to the development of preventive orthodontic and public health strategies [6,31,32]. This study aimed to assess oral hygiene behaviors, preventive oral health practices, and self-reported oral health experiences using the WHO Oral Health Questionnaire and to investigate their association with Angle malocclusion classes in children aged 6–9 years, assessed using digital intraoral scanning.

## 2. Materials and Methods

### 2.1. Subjects

A cross-sectional observational study was conducted. The study population was the schoolchildren of 6–9-year schools in the Pristina region in Kosovo (*n* = 90). From this population, a random sample was taken according to the “habitat” of residence (urban, rural). In total, 3 schools were selected, and the sample size was 30 from each school. Each school was informed by the Municipal Directorate of Education of Prishtina about the study to be conducted.

#### 2.1.1. Inclusion and Exclusion Criteria

Inclusion criteria: children aged 6–9, presence of mixed and permanent dentition with malocclusion, no previous orthodontic treatment, and no orthodontic treatment in progress. Exclusion criteria: Craniofacial anomalies or syndromes, history of maxillofacial surgery or trauma, systemic conditions affecting craniofacial growth, or incomplete or poor-quality 3D scans.

#### 2.1.2. Sample Size Calculation

The calculation of the sample size was performed using an online sample size determination tool for the two-sample *t*-test, assuming a medium effect size (Cohen’s d = 0.5), with probability (α = 0.05) and a statistical power of 90% (1 − β = 0.90) [33]. Based on these parameters, it was determined that a minimum of 86 participants were required. To increase the robustness of the analyses and to account for possible data loss or unusable 3D scans, the final sample size was rounded to 90 participants. Because no prior data were available regarding expected differences between Angle malocclusion groups, a medium effect size (Cohen’s d = 0.5) was assumed for sample size estimation to ensure adequate statistical power.

### 2.2. Ethical Considerations

The study protocol was reviewed and approved by the Ethics Committee for Scientific Research of the Faculty of Medicine in Prishtina with reference number 489 before the start of the study. All procedures were performed in accordance with the ethical standards of the Declaration of Helsinki for medical research involving human subjects. Parents or legal guardians received detailed oral and written information regarding the objectives and methodology of the study before participation. Written informed consent was obtained from the parents or legal guardians of all children included in the study. Confidentiality and anonymity of participant data were strictly maintained throughout the study and publication process.

### 2.3. Questionnaire

For children aged 6–9, the closest current WHO questionnaire tool is the generic WHO Oral Health Questionnaire for Children, 2013, in Annex 8 of Oral Health Surveys: Basic Methods, 5th edition [34].

During the first weeks, the criteria for diagnosis and questionnaire completion were calibrated in 3 schools. The dentist in each team performed the physical examination, and the assistant assisted in completing the questionnaire. Data collection was carried out during 2026, after coordinating the data collection days with the management of each school and, in all cases, with prior written authorization from the parents or guardians of each child. The questionnaire collected sociodemographic information that could be related to motivation for oral health, such as parents’ educational level and residential habitat (urban or rural). Children were asked how they rated their dental and gum health (excellent, very good, good, average, poor, very poor): Questions were also included regarding how often, occasionally, rarely, never they had experienced a toothache or oral discomfort during the past 12 months. They were also asked about their visit to the dentist in the past 12 months, when was the last time they had visited a dentist (less than 3 months ago, between 3 months and a year ago, more than a year ago, or never), and the reason for this visit (check-up, pain, treatments), the reason for the last visit to the dentist. Other questions related to oral health care practices, such as how often they brushed their teeth (regularly or not, and if so, how many times a day), whether they had an electric toothbrush, whether they used dental floss, and whether they used fluoride toothpaste. There were also questions directly related to the following problems: how satisfied they are with the condition of their teeth, how often they avoid smiling, whether their friends made fun of their teeth, whether pain or discomfort has forced them to miss school, difficulty biting hard foods, difficulty chewing: then how often they consumed several times a day, every day, several times a week, once a week, several times a month, never foods such as fresh fruit, and what level of education their parents had completed.

### 2.4. Malocclusion Classification

**Figure 1:**
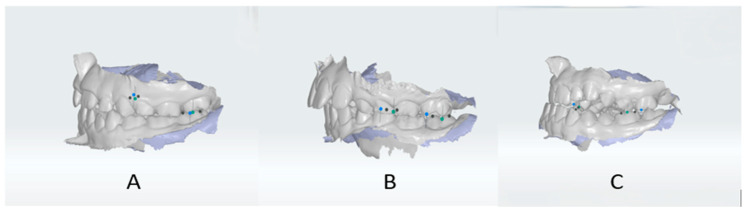
(**A**–**C**) Representative screenshots from the SHINING 3D software (Version 3.6.0.7) illustrating Angle’s classification of malocclusion based on digital intraoral scans: (**A**) Class I malocclusion, (**B**) Class II malocclusion, and (**C**) Class III malocclusion.

Digital intraoral scans were obtained using an Aoralscan 3 intraoral scanner (SHINNING 3D Technology Co., Ltd., Hangzhou, China) [35]. The digital models were used exclusively for the assessment and classification of malocclusion according to Angle’s classification (Class I, Class II, and Class III) (Figure 1A–C). Classification was based on the sagittal relationship of the first permanent molars. Class I malocclusion was defined as a normal molar relationship in which the mesiobuccal cusp of the maxillary first molar occludes with the mesiobuccal groove of the mandibular first molar, maybe with crowding, spacing, or other dental anomalies. Class II malocclusion was defined as a distal relationship between the mandibular and maxillary first molars in which the maxillary first molar’s mesiobuccal cusp is positioned anterior to the mandibular first molar’s mesiobuccal groove. Class III malocclusion was defined as a mesial relationship between the mandibular and maxillary first molars, in which the mesiobuccal cusp of the maxillary first molar was positioned posterior to the mesiobuccal groove of the mandibular first molar [36].

#### 2.4.1. Examiner Calibration and Reliability Assessment

Before the start of the study, the orthodontist responsible for malocclusion assessment underwent a calibration process using anonymized digital intraoral scans representing all three Angle malocclusion classes. Intra-examiner reliability was evaluated by reclassifying a subset of scans after a one-week interval. Inter-examiner reliability was assessed by having a second orthodontist independently evaluate the same set of scans.

Cohen’s kappa coefficients were calculated for both intra-examiner and inter-examiner agreement. The intra-examiner Cohen’s kappa coefficient was 0.818, and the inter-examiner Cohen’s kappa coefficient was 0.813, indicating excellent agreement. All malocclusion classifications were based on the sagittal relationship of the first permanent molars according to Angle’s classification. Digital intraoral scans were reviewed at two separate time points to ensure consistency and repeatability of the assessments.

#### 2.4.2. Construction of the Q10 Composite Score

The Q10 section initially consisted of six items related to oral-health-related quality of life and psychosocial impact. Following item-total correlation analysis, items Q10(b) and Q10(c) demonstrated weak correlations and were excluded from the final composite score. Consequently, the final score was calculated using Q10(a), Q10(d), Q10(e), and Q10(f).

Responses were coded dichotomously (Yes = 1; No = 0; “Don’t know” = 0). Individual item scores were summed to obtain the total Q10 score, and mean scores were calculated by dividing the total score by the number of included items. Higher scores indicated greater oral-health-related impact.

### 2.5. Statistical Analysis

Descriptive statistics (means ± standard deviation for continuous variables; frequencies and percentages for categorical variables) were computed to summarise participants’ characteristics and questionnaire responses. Associations between Angle malocclusion classes and categorical variables from the WHO Oral Health Questionnaire were tested using Chi-square (χ^2^) tests. When more than 20% of expected cell counts were below 5, categories were combined, or Fisher’s exact test was used to ensure valid inference. Spearman’s rank correlation coefficient was calculated to evaluate relationships between ordinal variables (e.g., age and Q10 scores). Internal consistency of the Q10 scale was assessed using Cronbach’s α. Statistical significance was set at α = 0.05, and analyses were performed using SPSS Statistics 23.0 (IBM Corp., Armonk, NY, USA) and Statistica 7.1 (StatSoft Inc., Tusla, OK, USA). Because multiple Chi-square tests were conducted, we acknowledge that the family-wise error rate increases; therefore, results should be interpreted as exploratory and with caution.

## 3. Results

A total of 90 children aged 6–9 years were included in the study. According to Angle classification, 21 children (23.3%) were classified as Class I, 61 (67.8%) as Class II, and 8 (8.9%) as Class III malocclusion. Based on sex distribution, 42 (46.7%) were boys, and 48 (53.3%) were girls. Regarding self-perceived oral health, most participants evaluated the condition of their teeth and gums as “good” or “average.” For dental health assessment, 34 children (37.8%) reported “good” dental health, while 32 (35.6%) reported “average” dental health. Similar findings were observed for gingival health. Concerning oral symptoms, 30 children (33.3%) reported occasional toothache or discomfort during the previous 12 months, whereas 24 (26.7%) reported never experiencing a toothache. Dental attendance patterns showed that most children had visited the dentist at least once during the previous 12 months. The most common reason for the last dental visit was pain, reported by 49 children (54.4%), followed by routine dental check-ups in 21 children (23.3%). Regarding oral hygiene practices, 42 children (46.7%) reported brushing their teeth two or more times per day, while 21 children (23.3%) brushed once daily. All participants reported using a toothbrush and toothpaste for oral hygiene maintenance. However, awareness regarding fluoride-containing toothpaste was limited, as all children answered “don’t know” when asked whether their toothpaste contained fluoride. In the oral-health-related quality-of-life section (Q10), 21 children (23.3%) reported dissatisfaction with the appearance of their teeth, while 24 children (26.7%) reported avoiding smiling or laughing because of their teeth. Additionally, 31 children (34.4%) experienced difficulty biting hard foods, and 14 children (15.6%) reported school absence due to dental pain or discomfort. Fresh fruit consumption was reported frequently among participants, with 40 children (44.4%) consuming fresh fruits daily and 28 children (31.1%) consuming them several times per week. Regarding parental educational level, most fathers had completed high school education (45.6%), while 26.7% had completed college or university education. Similarly, most mothers had completed high school education (51.1%), followed by college or university education (28.9%) (Table 1).

### 3.1. Reliability Analysis of Q10

The Q10 section of the questionnaire consisted of six items related to oral-health-related quality of life and psychosocial impact. Reliability analysis demonstrated a Cronbach’s Alpha value of 0.500, indicating relatively low internal consistency between questionnaire items. Corrected item-total correlation analysis demonstrated weak correlations for Q10(b) (0.297) and Q10(c) (0.043), suggesting that these items contributed less strongly to the composite score compared with the remaining questionnaire items (Table 2).

Table 3 shows the corrected values of the total Cronbach’s Alpha for each item/question of the questionnaire related to Q10. Since the correlation for Q10(b) (0.297), Q10(c) (0.043) is weak (De Vaus suggests that a correlation less than 0.30 is a weak correlation/Surveys in Social Research, Routledge, p. 184), then these items/questions are not used to form composite scores.

### 3.2. Descriptive Statistics of Q10 Total and Mean Scores

Descriptive statistical analysis showed that the total Q10 score ranged from 4 to 21, with a mean value of 7.60 ± 2.44 and a median value of 8. The average score (Mean) ranged from 1.00 to 5.25, with a mean value of 1.90 ± 0.61 (Table 4).

**Figure 2:**
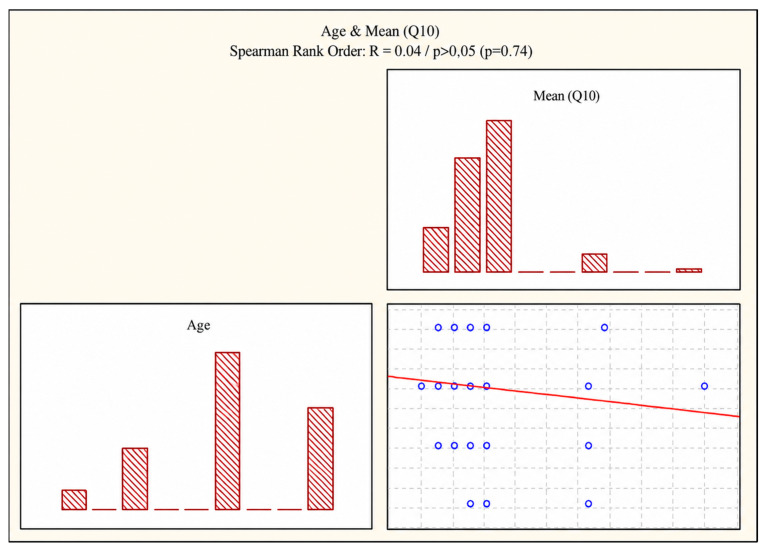
Correlation between Age and Mean Q10 Score.

Spearman’s correlation analysis showed a very weak, statistically insignificant positive association between age and the value of the average score related to children’s problems (Q10) (R = 0.04; *p* = 0.70) (Figure 2).

### 3.3. Association Between Angle Malocclusion Classes and WHO Questionnaire Variables

Chi-square (χ^2^) analysis was performed to evaluate the association between Angle malocclusion classes and WHO Oral Health Questionnaire variables (Q1–Q14). No statistically significant associations were identified between malocclusion classes and questionnaire responses (*p* > 0.05).

The highest χ^2^ value was observed for Q7 (frequency of tooth cleaning) (χ^2^ = 11.97; *p* = 0.152), followed by Q3Teeth and Q3Gums (χ^2^ = 8.92; *p* = 0.540). Questions related to oral-health-related quality of life, including dissatisfaction with dental appearance (Q10.1), avoidance of smiling (Q10.2), teasing by peers (Q10.3), school absence due to dental pain (Q10.4), and chewing difficulties (Q10.5–Q10.6), also showed no statistically significant association with malocclusion classes (Table 5).

Overall, the findings suggest that oral hygiene behaviors, preventive oral health practices, dietary habits, and oral-health-related experiences were relatively similar among children with Class I, Class II, and Class III malocclusion.

## 4. Discussion

The present study found no statistically significant associations between Angle malocclusion classes and any WHO Oral Health Questionnaire variables, indicating that oral hygiene behaviours and oral-health-related experiences were similar across malocclusion classes. Similar findings have been reported in previous pediatric oral health studies, where oral hygiene behaviors and oral-health-related quality of life were shown to be influenced more strongly by behavioral and socioeconomic factors than by malocclusion classification alone [37,38]. The absence of statistically significant differences among malocclusion groups may be explained by the multifactorial nature of oral hygiene behaviors during childhood. Oral hygiene practices in children are strongly associated with parental supervision, oral health education, dietary habits, and access to preventive dental care. Çelikel et al. reported that children’s oral hygiene knowledge does not always correspond with actual oral hygiene behavior, emphasizing the importance of environmental and educational influences on pediatric oral health practices [39]. Although Q7 (frequency of tooth cleaning) had the largest χ^2^ statistic among the variables tested, the association remained non-significant, indicating no evidence of differing tooth-cleaning routines between malocclusion classes. Previous literature suggests that irregular toothbrushing habits and inadequate oral hygiene may contribute to plaque accumulation and increased oral health complications in children presenting with malocclusions [40,41]. Questions related to oral-health-related quality of life and psychosocial impact (Q10 section) also did not demonstrate statistically significant associations with Angle classes. Nevertheless, a proportion of children reported dissatisfaction with dental appearance, avoidance of smiling, teasing by peers, and functional difficulties related to chewing and biting. These findings are consistent with studies demonstrating that malocclusion may negatively affect children’s psychosocial well-being and oral-health-related quality of life even in younger age groups. Daneshvar et al. demonstrated that malocclusion may contribute to dissatisfaction with dental appearance and impaired oral function in schoolchildren [42]. Similar findings were reported by Sunal Aktürk et al., who investigated the impact of Angle malocclusion classes on oral-health-related quality of life in adolescents. The authors observed no statistically significant differences between Class I, II, and III malocclusion groups regarding overall CPQ11–14 scores, except for the social well-being domain, where Class III patients demonstrated greater psychosocial impact. These findings are partially consistent with the present study, where psychosocial concerns related to dental appearance were observed despite the absence of statistically significant differences between malocclusion classes [43]. Chiba et al., in a systematic review and meta-analysis, concluded that malocclusions may negatively influence oral-health-related quality of life in children and adolescents, particularly regarding emotional and social well-being. However, the authors also emphasized that the magnitude of this effect varies according to age, malocclusion severity, and psychosocial maturity. This may explain why the present study, which included younger children aged 6–9 years, demonstrated relatively limited psychosocial differences between malocclusion groups [44]. Tondolo Junior et al., in a seven-year cohort study, reported that children with malocclusion may experience poorer oral-health-related quality of life compared with children without malocclusion, especially in cases involving anterior irregularities and increased overjet. The authors further suggested that the psychosocial and functional consequences of malocclusion may persist throughout growth and adolescence. These observations support the present findings, where a proportion of children reported dissatisfaction with dental appearance and difficulties related to chewing and biting despite the absence of statistically significant intergroup differences [45]. Recent literature has additionally demonstrated that malocclusion-related quality-of-life impairment may become more evident with increasing malocclusion severity. A recent cross-sectional study published in 2025 reported that deterioration in children’s quality of life was positively correlated with the severity of malocclusion, particularly regarding emotional well-being and caregiver perception. Nevertheless, the overall psychosocial impact in younger pediatric populations was generally described as mild to moderate, which is in agreement with the current findings [46]. Pan et al. also stated that the prevalence of malocclusion and its psychosocial effects can vary based on age and gender, reinforcing the idea that quality of life in relation to oral health is a multidimensional issue, shaped by clinico-behavioral aspects. This supports the notion that malocclusion per se may not adequately account for oral hygiene behavior and psychosocial consequences in pediatric groups [47]. One other significant finding of the present study was low levels of awareness concerning fluoride-containing toothpaste. While all participants indicated use of toothbrushes and toothpaste, most children responded “don’t know” when asked whether their toothpaste contained fluoride. This finding may indicate an inadequate preventive oral health education amongst children and parents. Prior research noted that knowledge about preventive oral hygiene among children is inconsistent, despite regular toothbrushing practices [48]. Because the present study included children aged 6–9 years, this finding should be interpreted with caution. Children in this age group may not be fully aware of the fluoride content of the toothpaste used at home, and their responses may reflect limited knowledge rather than the actual absence of fluoride use. Therefore, parental knowledge and supervision may provide a more reliable indicator of preventive oral health awareness in young children. Future studies should consider including caregiver questionnaires to obtain more comprehensive information regarding fluoride-related knowledge and practices. There are a number of limitations in the current work. The cross-sectional design does not permit induction of any causal link between oral hygiene behaviors and malocclusion classes. Further, the relatively small number of children with Class III malocclusion may have limited statistical power for the discovery of patterns of intergroup differences. In addition, the regional pediatric sample from Kosovo was the population of this study, which may affect the generalizability of the findings. Nevertheless, this study does yield important epidemiological evidence of oral hygiene behaviors and experiences with oral-health issues in children with different Angle malocclusion classes. The use of the WHO standardized questionnaire is an important methodological advantage, facilitating comparison with other international studies of pediatric oral health, and will be conducive to developing childhood preventive oral health and orthodontic interventions. Furthermore, although statistical significance was assessed using Chi-square tests, the absence of effect size estimates and confidence intervals may limit the interpretation of the magnitude and clinical relevance of the observed associations. Future studies with larger samples should incorporate these measures to provide more comprehensive statistical interpretation.

### Limitations

The relatively uneven distribution of malocclusion groups, particularly the smaller number of Class III participants, may have reduced the statistical sensitivity for detecting intergroup differences. A further limitation of the existing study is the unequal distribution of participants among the Angle malocclusion groups. Overall, we found that the children who belonged to the Class III malocclusion group had a reduced number of individuals compared to those from Class I and Class II groups. Such an imbalance might have reduced the statistical power to identify small or moderate disparities amongst the groups and introduced the risk of a Type II error. Consequently, the absence of statistically significant associations should not be interpreted as definitive evidence of absence of association, since insufficient statistical power may have limited the ability to detect subtle differences between groups. Therefore, the lack of statistically significant associations should not necessarily be considered as an indication of the absence of an actual relationship between oral hygiene behaviour and the type of malocclusion. More extensive studies with more balanced distributions of subjects are required to make the conclusions more robust. Thus, future longitudinal studies in larger, more balanced groups of malocclusion are recommended in order to examine possible associations between oral hygiene, psychosocial effects on children, and craniofacial development during childhood.

Another methodological limitation concerns the sample size calculation. During the planning phase, the sample size was estimated using a two-sample *t*-test. However, the final analyses relied primarily on Chi-square tests, and therefore, the sample size calculation was not fully aligned with the statistical methodology ultimately used. This inconsistency should be considered an important methodological weakness and may have influenced the ability of the study to detect subtle associations. Future studies should employ power analyses specifically designed for categorical outcomes and Chi-square-based comparisons.

## 5. Conclusions

Within the limitations of this cross-sectional study, oral hygiene behaviors and oral-health-related experiences appeared relatively similar among children with different Angle malocclusion classes. Although no statistically significant associations were identified, psychosocial concerns and limited preventive oral-health awareness were observed in a proportion of participants. The WHO Oral Health Questionnaire may represent a valuable epidemiological tool for pediatric oral-health and orthodontic screening programs. Despite the absence of statistically significant intergroup differences, several clinically relevant observations emerged. A considerable proportion of children reported irregular oral hygiene and low awareness of fluoride-containing toothpaste, dissatisfaction with dental appearance, and trouble with chewing and biting. This finding demonstrates the significance of early preventive oral health education and the importance of better parental and school oral health promotion programmes in the mixed dentition age. The WHO Oral Health Questionnaire was found to be an accurate, valuable epidemiological tool used to evaluate oral hygiene behaviors, oral health-related quality of life, and preventive oral health concepts in children. Importantly, the present results provide the key epidemiological baseline of pediatric oral health behaviors and malocclusion-related experiences in children from Kosovo.

## Figures and Tables

**Table 1:** Children’s Oral Health Questionnaire (WHO Standard).

Questions						N (%)
Q1 Sex						
Boy						42 (46.7%)
Girl						48 (53.3%)
Q2 How would you describe the health of your teeth and gums?						
Q2Teeth						
Excellent						8 (8.9%)
Very good						9 (10.0%)
Good						34 (37.8%)
Average						32 (35.6%)
Poor						3 (3.3%)
Very poor						4 (4.4%)
Q2Gums						
Excellent						8 (8.9%)
Very good						9 (10.0%)
Good						34 (37.8%)
Average						32 (35.6%)
Poor						3 (3.3%)
Don’t know						4 (4.4%)
Q3 How often during the past 12 months did you have a toothache or feel discomfort due to your teeth?						
Often						9 (10.0%)
Occasionally						30 (33.3%)
Rarely						20 (22.2%)
Never						24 (26.7%)
Don’t know						7 (7.8%)
Q4 How often did you go to the dentist during the past 12 months?						
Once						12 (13.3%)
Twice						22 (24.4%)
Three times						19 (21.1%)
Four times						11 (12.2%)
More than four times						9 (10.0%)
I have not visited the dentist during the past 12 months						7 (7.8%)
I have never received dental care/visited a dentist						8 (8.9%)
I don’t know/don’t remember						2 (2.2%)
Q5 What was the reason for your last visit to the dentist?						
Pain						49 (54.4%)
Treatment						12 (13.3%)
Routine check-up						21 (23.3%)
Not applicable, did not visit the dentist						8 (8.9%)
Q6 How often do you clean your teeth?						
Several times a month (2–3 times)						11 (12.2%)
Once a week						4 (4.4%)
Several times a week (2–6 times)						12 (13.3%)
Once a day						21 (23.3%)
2 or more times a day						42 (46.7%)
Q7 Do you use any of the following to clean your teeth or gums?						
Toothbrush						
No						0 (0.0%)
Yes						90 (100.0%)
Q8 Do you use toothpaste to clean your teeth?						
No						0 (0.0%)
Yes						90 (100.0%)
Q9 Does the toothpaste you use contain fluoride						
No						0 (0.0%)
Yes						0 (0.0%)
Don’t know						90 (100.0%)
Q10a I am not satisfied with the appearance of my teeth						
Don’t know						4 (4.4%)
Yes						21 (23.3%)
No						65 (72.2%)
Q10b I often avoid smiling or laughing because of my teeth						
No						64 (71.1%)
Yes						24 (26.7%)
Don’t know						2 (2.2%)
Q10c Other children tease me about my teeth						
No						69 (76.7%)
Yes						21 (23.3%)
Don’t know						0 (0.0%)
Q10d Pain or discomfort from my teeth has caused me to miss school or stay home for full days						
No						76 (84.4%)
Yes						14 (15.6%)
Don’t know						0 (0.0%)
Q10e I have difficulty biting hard foods						
No						58 (64.4%)
Yes						31 (34.4%)
Don’t know						1 (1.1%)
Q10f I have difficulty chewing food						
No						2 (2.2%)
Yes						19 (21.1%)
Don’t know						79 (76.7%)
Q11 How often does the child consume any of the following foods or drinks?						
Fresh fruits						
Never						0 (0.0%)
Several times a month						1 (1.1%)
Once a week						10 (11.1%)
Several times a week						28 (31.1%)
Every day						40 (44.4%)
Several times a day						11 (12.2%)
Q12 What level of education has your father completed?						
Primary school						1 (1.1%)
Secondary school						24 (26.6%)
High school						41 (45.6%)
College/university						24 (26.7%)
Q13 What level of education has your mother completed?						
Primary school						7 (7.8%)
Secondary school						11 (12.2%)
High school						46 (51.1%)
College/university						26 (28.9%)

**Table 2:** Reliability analysis of the Q10 section of the questionnaire.

Cronbach’s Alpha	N of Items
0.500	6

**Table 3:** Cronbach’s Alpha values for each item related to Q10.

	Scale Mean If Item Deleted	Scale Variance If Item Deleted	Corrected Item-Total Correlation	Cronbach’s Alpha If Item Deleted
Q10(a)	8.68	1.839	0.144	0.523
Q10(b)	8.67	1.708	(0.297)	0.431
Q10(c)	8.59	2.132	(0.043)	0.547
Q10(d)	8.51	1.916	0.315	0.436
Q10(e)	8.72	1.619	0.380	0.382
Q10(f)	8.61	1.634	0.398	0.375

**Table 4:** Descriptive Statistics for Q10 Scores.

Variable	Valid N	Mean	Confidence −95.00%	Confidence +95.00%	Median	Minimum	Maximum	Std. Dev.
Total	90	7.60	7.09	8.11	8	4	21	2.44
Mean	90	1.90	1.77	2.03	2.00	1.00	5.25	0.61

**Table 5:** Association between Angle malocclusion classes and WHO Oral Health Questionnaire variables.

Questionnaire Variable	χ^2^	df	*p*-Value	Interpretation
Q1	0.18	2	0.915	Not significant
Q2	5.77	6	0.450	Not significant
Q3Teeth	8.92	10	0.540	Not significant
Q3Gums	8.92	10	0.540	Not significant
Q4	4.59	8	0.800	Not significant
Q5	4.80	8	0.778	Not significant
Q6	1.38	4	0.848	Not significant
Q7	11.97	8	0.152	Not significant
Q8	6.22	8	0.623	Not significant
Q9a	0.47	2	0.790	Not significant
Q9b	2.61	2	0.271	Not significant
Q10.1	5.53	4	0.237	Not significant
Q10.2	6.31	4	0.177	Not significant
Q10.3	2.86	4	0.582	Not significant
Q10.4	2.70	2	0.258	Not significant
Q10.5	4.44	4	0.350	Not significant
Q10.6	1.97	4	0.742	Not significant
Q11	2.83	4	0.587	Not significant
Q12	2.57	2	0.277	Not significant
Q13	3.39	4	0.495	Not significant

## Data Availability

The data associated with this study are not publicly available due to ethical and privacy restrictions involving pediatric participants. The dataset contains clinical and questionnaire-based information collected from children, and public data sharing was not included within the scope of parental informed consent approved by the Ethics Committee. Therefore, the data are available from the corresponding author upon reasonable request and in accordance with institutional ethical regulations.
